# Pictorial representation of attachment: measuring the parent-fetus relationship in expectant mothers and fathers

**DOI:** 10.1186/1471-2393-13-138

**Published:** 2013-06-27

**Authors:** Hedwig JA van Bakel, A Janneke BM Maas, Charlotte MJM Vreeswijk, Ad JJM Vingerhoets

**Affiliations:** 1 Tilburg School of Social and Behavioral Sciences, Tilburg University, Warandelaan 2, P.O. Box 90153, Tilburg, 5000 LE, the Netherlands; 2 Centre for Infant Mental Health, Dimence, Deventer, the Netherlands; 3 Herlaarhof, Centre for Child and Adolescent Psychiatry, Vught, the Netherlands

**Keywords:** Antenatal, Attachment, Mother, Father, Pregnancy, Fetus

## Abstract

**Background:**

Over the past decades, attachment research has predominantly focused on the attachment relationship that infants develop with their parents or that adults had with their own parents. Far less is known about the development of feelings of attachment in parents towards their children. The present study examined a) whether a simple non-verbal (i.e., pictorial) measure of attachment (Pictorial Representation of Attachment Measure: PRAM) is a valid instrument to assess parental representations of the antenatal relationship with the fetus in expectant women and men and b) whether factors such as gender of the parent, parity, and age are systematically related to parental bonding during pregnancy.

**Methods:**

At 26 weeks gestational age, 352 primi- or multiparous pregnant women and 268 partners from a community based sample filled in the PRAM and the M/PAAS (Maternal/Paternal Antenatal Attachment Scale, Condon, 1985/1993).

**Results:**

Results show that the PRAM was significantly positively associated to a self-report questionnaire of antenatal attachment in both expectant mothers and fathers. Age and parity were both found significantly related to M/PAAS and PRAM scores.

**Conclusions:**

The present findings provide support that the PRAM is as a valid, quick, and easy-to-administer instrument of parent-infant bonding. However, further research focusing on its capacity as a screening instrument (to identify parents with serious bonding problems) and its sensitivity to change (necessary for the use in evaluation of intervention studies) is needed, in order to prove its clinical value.

## Background

Over the past decades, attachment research has largely focused on the quality of the attachment relationship that infants develop in the course of the first year of life with their parents, in particular the mother [1]. Also studies and knowledge about the representations of attachment relationships that adolescents or adults have of their own parents are increasing [2]. Far less is known about the development of feelings of attachment in parents *towards* their children. Klaus and Kennell [3] introduced the term ‘maternal bonding’ and defined the bond between mothers and their newborns as a biologically based emotional investment in the infant. Maternal emotional investment in the infant, however, does not start from birth onwards. It is recognized that this bond already starts to develop before birth [4-6] and that building a bond or ‘relationship’ with the unborn child is a key developmental task for a successful psychological adjustment of pregnant women [7].

Prenatal attachment can be described as the parent’s emotions, perceptions, and behaviors that are related to the fetus. These unique emotions and behaviors may be represented by an affiliation and interaction with the unborn baby and the desire of the parent to know and to be with the unborn baby [8]. Analysis of the literature suggests that prenatal attachment during pregnancy is not the same kind of attachment relationship as Bowlby [9] and Ainsworth et al. [10] have defined it, but a multi-faceted construct guided by the caregiving system [11]. Although attachment behaviors are essentially about eliciting care from others, the substance of maternal–fetal relationships deals with the development of feelings of parental love and protection. These relationships can be seen as a strong emotional tie. Therefore it seems reasonable to refer to them as attachment, as a generation of researchers has done before [4,11-13]. The extent to which women feel attached and emotionally connected to the unborn child has been shown to be related to various pre- and postnatal parent and child outcomes, such as feelings of competence in infant feeding and care [14] parental mood [15] and the quality of mother’s interaction with the infant after birth [16].

Various self-report questionnaires have been developed to assess the quality of the mother-fetus relationship or antenatal attachment. The Maternal Fetal Attachment Scale (MFAS: [12], the Prenatal Attachment Inventory (PAI: [13]), and the Maternal Antenatal Attachment Scale (MAAS: [4]) are among the most frequently used scales which all tap overt (i.e., conscious) thoughts, feelings, attitudes and behaviors of the parents towards the fetus. All three questionnaires proved to be psychometrically sound instruments in Caucasian middle-class samples [17]. For research purposes -aimed at clarifying and identifying underlying mechanisms- these questionnaires were proven to be valid and reliable. For routine use in clinical settings, however, these measures are less feasible, because they require a certain reading level and more time to complete. In addition, it is unclear to what extent the answers are influenced by social desirability. Earlier studies revealed a tendency of pregnant women to respond in a socially desirable manner on questionnaires about mother-fetus relationships [18,19]. In clinical settings there are several examples of simple, often non-verbal instruments, which can easily and quickly be applied to obtain information for the clinician, such as pain and wellbeing measures, and a pictorial measure of suffering [20]. We here propose an alternative, non-verbal tool that is fast and easy for expectant parents to complete, and which can be easily administered at antenatal routine visits by obstetricians and midwives.

The Pictorial Representation of Attachment Measure (PRAM: [21]) is a tool designed to measure parental representations of the (antenatal) parent-infant relationship in (expectant) fathers and mothers. It is inspired by the Pictorial Representation of Illness and Self Measure (PRISM) [20] a measure originally developed to assess the burden of suffering due to illness into a patient’s life, and the Inclusion of Other in the Self Scale (IOS: [22]) a single-item non-verbal measure for the structure of interpersonal closeness. The PRISM requires individuals or patients to put a disk representing their illness in relation to a disk representing the self. The distance between the self and the other (‘illness’) disk is measured and represents the impact of the illness on the self. Various studies have demonstrated the reliability, validity and clinical and research usefulness of the original PRISM or adapted versions of this tool in patients with cancer [23], dermatological problems [24], chronic pain [25], psoriasis, and rheumatic arthritis [20]. A recent study also showed the validity of the PRISM as a method for the assessment of suffering after trauma in PTSD patients [26]. In addition, one previous study has used this instrument to assess suffering in a non-physical-illness condition [27,28]. Parents having experienced the loss of a premature child were asked to indicate the place of their lost child in their lives. The results of that study -using an adapted paper-and-pencil version of the PRISM- showed that a shorter distance between the ‘self’ circle and a cross representing the child, correlated significantly with greater grief due to loss of the child.

The PRAM also shows a similarity with pictorial measures designed to assess interpersonal relationships [23,29] and attachment networks in adults [30], which have shown convergent validity. We here introduce the PRAM and expect it to be a useful tool to determine the place of the (unborn) child in the lives in (expectant) parents. A preliminary study with 76–80 participants showed promising results [21].

Until now, research on prenatal attachment has mainly focused on expectant mothers, rather than on fathers. Nevertheless, not only mothers but also fathers may feel more or less attached or connected to their unborn child [31], since for them also, pregnancy is a time of psychological preparation. Condon [32] described father-fetus bonding as a subjective feeling of love for the unborn child, which is at the heart of a man’s experience of early parenting.

Although one might assume that the way women and men interact with the unborn child may differ, evidence demonstrating gender differences in feelings of antenatal attachment is not consistent. Studies comparing maternal and paternal antenatal bonding show contradictory results with some studies showing mothers to have more and stronger feelings of bonding towards the fetus compared to their partners [33-35] whereas other studies failed to demonstrate such differences [36] or even yielded opposite findings [37]. Moreover, only few studies have evaluated both the feelings of antenatal attachment in women and their partner concurrently [34,35].

Studies on prenatal attachment as experienced and perceived by primiparous and multiparous pregnant women and their partners have also yielded inconsistent results. Cranley [12] failed to find a relationship between prenatal attachment and parity, whereas Ferketich and Mercer [38] and Van Bussel, Spitz, and Demyttenaere [18] showed that multiparous women had lower attachment scores compared to primiparous women. Sorensen and Schuelke [39] further demonstrated that prenatal fantasies about the unborn child were more prevalent in primiparae than in multiparae. However, there is a lack of studies comparing parents expecting a first child compared to those already having a child with respect to the felt relationship with the fetus [40]. Finally, there is some evidence that demographic variables such as age of the parent show a negative relationship with feelings of attachment, although these findings are not consistent [12,35,41-43]. A recent study by McMahon et al. [44] showed that women having their first baby at an older age, appear to have some psychological advantages over younger women, because they are more resilient and better adjusted. This may result in less preoccupation with the fetus (i.e., as reflected in lower maternal-fetal attachment scores). Although the reasons for inconsistent results are not always clear, the fact that maternal antenatal attachment was measured during different time periods of pregnancy, with different instruments -each stressing slightly other aspects of the prenatal attachment relationship- in pregnant women whose ages varied across the different samples, may have played a role.

This study is the first to examine whether a newly developed non-verbal (pictorial) measure of parental representations of the antenatal relationship with the fetus relates to a widely used valid and reliable verbal self-report measure of parental representations of the antenatal relationship. If the PRAM is measuring feelings of bonding and connectedness, it should be associated significantly and meaningfully with self-report measures of antenatal attachment such as the Maternal Antenatal Attachment Scale (MAAS: [4]). The MAAS consists of items all focusing upon feelings, attitudes and behaviors towards the fetus, in contrast to scales such as the MFAS and PAI, which contain a number of items related to the pregnancy state and motherhood role. Moreover, Condon [32] also developed a paternal antenatal attachment scale (PAAS) similar to the MAAS. The PRAM specifically addresses the question “where do you place the baby in your life at this moment” and is therefore also specifically directed at the fetus per se. The present study focuses on (1) whether verbal and non-verbal (i.e., pictorial) representations of parent-fetus attachment are significantly correlated with each other and (2) whether gender of the parent, parity, and age are systematically connected with feelings of bonding or attachment as assessed by both the PRAM and verbal (MAAS and PAAS) measures.

## Methods

The present study is part of a larger prospective longitudinal project called ‘Expectant Parents’ on prenatal risk factors and postnatal infant development [45]. More details about recruitment of the participants and the data collection used in this study have been reported earlier [45] and are briefly summarized here. At the first routine antenatal visit (between 9–15 weeks gestational age) 835 women were invited to participate by their midwives, who informed them about the project and handed out a leaflet with written information about the aim and design of the study. Parents were asked to participate in a longitudinal study, in which parents were followed from pregnancy (15 weeks gestational age) until 24 months postpartum. The aim of the larger prospective study was to gain more insight into feelings, emotions, behaviors and expectations about the pregnancy and the fetus, and about experiences with and development of the child after birth. The purpose is to investigate the quality of parent-infant relationships from parents’ perspectives, both in the prenatal and postpartum period. This was also communicated to parents [45]. For the purpose of the present study we informed parents that we aimed to gain more insight into feelings and emotions with the pregnancy and with the developing fetus. Women with a poor understanding of Dutch or English language and those expecting multiple births were excluded from participation.

Three-hundred fifty two pregnant women and their partners (n=268, 78%) agreed to participate and gave their written consent. The mean age of the women was 31.5 years (SD=4.4), ranging from 17 to 44 years. The mean age of the partner was 34.0 years (SD=4.6), ranging from 22 to 49 years. The mean gestational age was 26 weeks (*SD* = 1; Range 23–30 weeks). Most participants were white Caucasian women with the Dutch nationality (84%). The other participants have another Western (7%) or non-western (9%) ethnic background (e.g., Polish, Moroccan, Turkish, Surinam or Asian). Both women and men had completed an average of 16 years of formal education (SD=4.1). Fifty-three percent of the women (n= 187) and 53% of the men (n=143) were primiparous. The Medical Ethical Committee of the Elisabeth Hospital Tilburg approved the study protocol.

### Procedure and measures

At 26 weeks gestational age parents were asked to fill in questionnaires (M/PAAS) and all participating women and their partners completed a pictorial measure during a home-visit (PRAM). Women and men who filled in the questionnaires (M/PAAS) after 31 weeks gestational age were excluded from the analyses to limit the gestational age range, since antenatal attachment is known to increase during the course of pregnancy with a peak towards the end of the third semester [46,47]. The M/PAAS and PRAM were administered in the same order. We first sent parents the M/PAAS at 24–26 weeks GA (by mail) and during the home-visit at 26 weeks GA parents completed the PRAM. At the home visit we collected all questionnaires that were filled out by the parents before the home-visit and were not send back to us. Our experience is that sending parents questionnaires before a home visit -with the instruction that we will collect the questionnaires at the home visit- improves the response rate. Due to this standard order we are not able to control for a possible effect of the M/PAAS on the PRAM.

#### Maternal antenatal attachment scale (MAAS)

All women completed the Maternal Antenatal Attachment Scale (MAAS: [4]; Dutch translation by Van Bussel et al., [18]) designed to assess the mother’s attachment with the unborn child. It consists of 19 items divided over two subscales: “Quality of Attachment (QA)” (11 items) and “Intensity of Preoccupation (IP)” (8 items). The subscale QA measures the quality of the mother’s affective experience towards the unborn child (such as feelings of closeness and tenderness versus feelings of detachment and distance or irritation). The second subscale IP assesses the strength of feelings toward the unborn child and the amount of time thinking or dreaming about and talking to the fetus. All items are scored on 5-point Likert scales. The minimum score for the Global Attachment (i.e., Total) MAAS (GA) score is 19 and the maximum score is 95. The scores of the subscale QA range between 11 and 55 and those of the subscale IP vary between 8 and 40. On both subscales and the GA, higher scores reflect a positive quality of attachment and a high preoccupation with the unborn child. Internal consistencies of the MAAS and its subscales were adequate in earlier research [4] as well as in the current study with Cronbach’s alpha’s of .76 (GA), .66 (QA) and .68 (IP).

#### Paternal antenatal attachment scale (PAAS)

The fathers completed the Paternal Antenatal Attachment Scale (PAAS: [4]; Dutch translation by Colpin, De Munter, Nys, & Vandemeulebroecke, [48]), which consists of 16 items and also contains the subscales “Quality of Attachment (QA)” (10 items) and “Intensity of Preoccupation (IP)” (6 items), which represent the same underlying dimensions as the maternal scales, i.e., the quality and the preoccupation of antenatal attachment. The 16 items are scored on a 5-point Likert scale. The minimum score for the Global Attachment (i.e., Total) PAAS score (GA) is 16 and the maximum score is 80. The scores of the subscale QA range between 10 and 50 and the score range of the scale IP is between 6 and 30. Higher scores reflect a positive quality of attachment and a high preoccupation with the fetus. Internal consistencies of the PAAS and its subscales were sufficient in the current study, with Cronbach’s alpha’s of .78 (GA), .61 (QA) and .65 (IP).

#### Pictorial representation of attachment measure (PRAM)

To assess the representation of antenatal attachment non-verbally, parents completed the PRAM [21]. The parent was provided with a white A4-size paper with a big circle in the centre (diameter of 18.6 cm), which represents the parent’s current life. A yellow circle of 5 cm in the centre of the big circle represents the parent her- or himself. The parent was handed a green round sticker (5 cm) and was asked to imagine that the green circle represents the unborn child. They were then asked "where would you place the baby in your life at the moment?". We also asked all parents to describe why they put the sticker at a particular distance. All parents were able to answer this question, indicating that there were no problems in understanding the instruction.

For quantative use, the main score is the PRAM Self-Baby-Distance (PRAM-SBD), i.e., the distance, in centimeters, between the centres of the 'Baby' and 'Self' circles. The PRAM-SBD could range from 0 to 9.30 cm. A smaller PRAM-SBD is regarded as indicating more feelings of attachment.

### Statistics

All statistical analyses were performed using SPSS 19.0 for Windows. Since all study variables (MAAS/PAAS GA, QA, IP and PRAM-SBD) were normally distributed, parametric methods were used. To explore the convergent validity of the PRAM, relationships between MAAS, PAAS, and PRAM-SBD, were computed with Pearson product–moment correlations. Pearson correlation coefficients and paired sample t-tests were performed to evaluate the scores of women and men on attachment measures and PRAM-SBD. In addition, analyses were conducted comparing women and men, and primi- and multiparous parents separately, and taking into account parental age.

## Results

### Concordance between verbal and non-verbal representations of parent-fetus attachment

To measure the convergent validity of the PRAM, we first analyzed the concordance between verbal and non-verbal measures of parent-fetus attachment. Table 1 summarizes the descriptive statistics of the MAAS, PAAS and PRAM-Self Baby Distance (PRAM-SBD) and the correlation coefficients (*r*) between these variables for women and men separately. The results show significant associations between PRAM-SBD, on the one hand, and MAAS and PAAS, on the other. The smaller the distance between Self and Baby, the higher the GA in both women and men (*r* = −.32 and *r* = −.44, respectively). Significant correlation coefficients were found for the subscales QA (*r* = −.23 in women and *r* = −.46, in men), and IP with PRAM-SBD (*r* = −.30 in women and *r* = −.33 in men). All correlations were significant at p < .001.

**Table 1:** Means and standard deviations of the study variables and Pearson correlation coefficients among MAAS, PAAS and PRAM-SBD variables

	**Women**				**Men**^**a**^			
	***M***	***SD***	***r***_***PRAM-SBD***_	**Range, Min -Max**	***M***	***SD***	***r***_***PRAM-SBD***_	**Range, Min -Max**
**Antenatal attachment**								
*Global Attachment*	75.38	6.27	-.32***	54-93	55.62	5.99	-.44***	36-71
*Quality Attachment*	49.88	3.30	-.23***	36-55	38.68	3.39	-.46***	25-46
*Intensity of Preoccupation*	25.50	3.92	-.30***	14-38	16.93	3.29	-.33**	9-26
**PRAM**								
*Self-Baby-Distance (SBD)*	3.66	1.86		0.0-8.40	4.56	2.07		.05-9.05

Note. ** p<.01, *** p<.001; N= between 243 and 352, ^**a**^ Means, SD’s and Min-Max for men (GA *M*=66.05, *SD*=7.12; Min-Max= 42.75-84.31; QA *M*=42.55, *SD*=3.73, Min-max=27.50-50.60; IP *M*=22.58, *SD*=4.39, Min-Max=12.00-34.67) corrected for different numbers of items between MAAS (19 items) and PAAS (16 items).

### Comparisons between mothers and fathers

Significant positive correlations were found between PRAM-SBD scores of partners (*r* = .27, p<.001). We further found significant positive associations between maternal and paternal MAAS and PAAS scores (GA, *r* [261] = .30, p<.001; QA, *r* [262] = .24, p<.001; IP, *r* [264] = .24, p<.001). Subsequent paired-samples *t*-tests revealed expectant mothers to score higher on GA, QA, and IP than expectant fathers (*t* = 19.47, p < .001; *t* = 26.97, p < .001; and *t* = 9.99, p < .001 respectively). In these analyses, we corrected for the different numbers of items in the M/PAAS. The mean PRAM-SBD was also significantly different for mothers and fathers (*t* = −4.85, *p* < .001) with fathers depicting a significantly larger distance than mothers (*M*_Father SBD_ = 4.56 cm, *SD* = 2.11; *M*_Mother SBD_ = 3.77 cm, *SD* = 1.87).

### Parity and related gender differences

**Figure 1:**
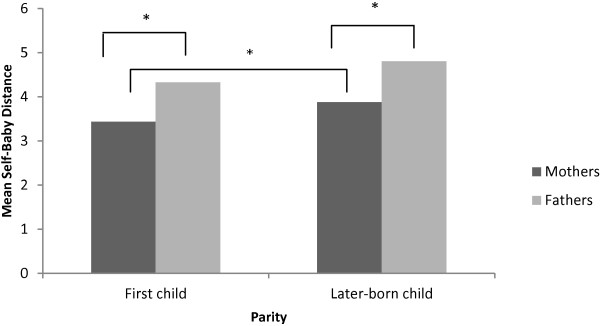
Mean self-baby distance of women and men on PRAM according to parity.

T-tests revealed significant differences between the mean PRAM-SBD scores of first time mothers (*M*_Mother SBD_ = 3.44 cm, *SD* = 1.82) and mothers already having a child (*M*_Mother SBD_ = 3.88 cm, *SD* = 1.90; *t*=2.01, *p* = .046) (Figure 1). For men, no significant differences were found between first time fathers (*M*_Father SBD_ = 4.33 cm, *SD* = 2.19) and fathers already having a child (*M*_Father SBD_ = 4.80 cm, *SD* = 1.94.; *t*=1.69, *p* = n.s).

**Figure 2:**
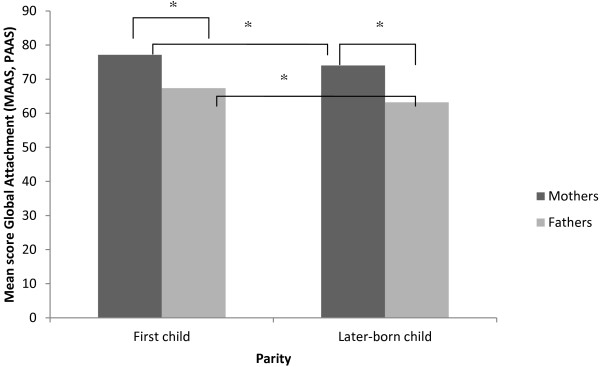
Mean Global Attachment of women and men on MAAS/PAAS according to parity.

Effects of parity were found on parents’ self-reported prenatal attachment on the MAAS and PAAS. Analyses showed that first time mothers reported significantly more GA (*t* = −4.78, *p* < .001), QA (*t = −*1.99, *p* = .047), and IP (*t* = −5.76, *p* < .001) than mothers already having a child*.* In men, also GA as well as both subscales were significantly different between first time fathers and fathers already having a child (GA *t* = −6.15, *p* < .001; QA *t* = −4.41, *p* < .001; IP *t* = −6.70, *p* < .001) (Figure 2).

In both primiparous (*r* = .24, *p* = .008) and multiparous (*r* = .29, *p* = .004) parents the correlation coefficients between partners on the PRAM-SBD were significantly correlated. Analyses on gender differences in parents expecting their first or later-born child showed similar results as those obtained from the whole sample. Both fathers expecting a first child and those already having a child showing a greater PRAM-SBD than mothers (*t* = −3.34, *p* = .001; *t* = −3.41, *p* = .001 respectively) (Figure 1).

This also holds for GA, QA and IP. Mothers expecting their first child had higher GA than fathers of first-borns (*M*_Mother GA_= 77.14, *SD* = 5.8; *M*_Father GA_= 67.37, *SD* = 6.85; *t* = 12.73, *p* < .001) (Figure 2). Similar results were found for QA and IP (*M*_Mother QA_ = 50.29, *SD* = 3.27; *M*_Father QA_ = 43.47, *SD* = 3.64; *t* = 19.49, *p* < .001; and *M*_Mother IP_ = 26.83, *SD* = 3.67; *M*_Father IP_ = 24.15, *SD* = 4.28; *t* = 5.92, *p* < .001). Comparable gender differences were found in parents of later-born children, with fathers reporting less feelings of attachment compared to mothers on the GA (Figure 2) and on the subscales QA and IP (*M*_Mother GA_= 74.00, *SD* = 6.43; *M*_Father GA_= 63.20, *SD* = 6.55; *t* = 15.18, *p* < .001; *M*_Mother QA_ = 49.48, *SD* = 3.47; *M*_Father QA_ = 41.43, *SD* = 3.64; *t* = 18.77, *p* < .001; *M*_Mother IP_ = 24.56, *SD* = 3.94; *M*_Father IP_ = 20.72, *SD* = 3.88; *t* = 8.69, *p* < .001).

### Parental age

Subsequent analyses on the association between parental age and antenatal attachment revealed significant associations. PRAM-SBD was found significantly positively related to both maternal and paternal age with Pearson coefficients of *r* (290) = .18 (*p* =.002) for women and *r* (221) = .17 (*p* =.009) for men. The younger the parent, the smaller the PRAM-SBD. Results with MAAS and PAAS data also showed that the younger the mother, the higher the reported feelings of GA (*r*[350] = −.30, *p* < .001), QA (*r*[350] = −.18, *p* = .001) and IP (*r*[352] = −.31, *p* < .001). Also younger fathers reported more feelings of GA (*r*[268]=−.18, *p =* .003) and more IP (*r*[270] = −.21, *p =* .001), whereas the association with QA did not reach significance (*r*[269] = −.12, *p =* .051).

## Discussion

The present study examined whether a simple non-verbal (i.e., pictorial) measure of attachment (Pictorial Representation of Attachment Measure: PRAM) is a valid and feasible instrument to measure parental representations of the antenatal relationship with the fetus in a non-clinical sample. Moreover, we examined gender, parity, and age effects.

PRAM-SBD was found to be significantly related to a self-report questionnaire of antenatal attachment in both expectant mothers and fathers. Results showed significant correlations for mothers and fathers between PRAM-SBD and the reported feelings of attachment as assessed with antenatal attachment questionnaires [4]. Parents who placed the sticker of the Baby closer to the Self also reported more feelings of attachment and more preoccupation with the fetus on a self-report questionnaire. The PRAM-SBD was related to all three dimensions of antenatal attachment (i.e., Global Attachment, Quality of Attachment and Intensity of Preoccupation) to the same extent. This suggests that the PRAM-SBD may be a reflection of a general feeling of bonding and connectedness with the unborn child at that moment.

Interestingly, significant positive relations were found between women and their partners for all study variables. This may suggest that at different time periods of pregnancy when mothers feel less or more attached with the fetus their partners also show less or more attachment behaviors towards the fetus. However, when comparing the mean scores of the pregnant women and their partners, the maternal attachment scores on the questionnaire were significantly higher and the PRAM-SBD was smaller than the scores of their partners on the questionnaires and the reported paternal PRAM-SBD. This may be a result of women experiencing the physical signs of pregnancy more directly. Quickening (i.e., the moment that refers to the initial motion of the fetus in the uterus as it is perceived or felt by the pregnant woman) also enhances feelings of attachment towards the fetus and may be more eminent to the mother than to her partner. Condon [32] demonstrated that fathers and mothers are nearly equivalent in their thoughts and feelings about the unborn child, but that fathers are less likely to express these feelings in behavioral ways. The use of pictorial measures, like the PRAM, might have diminished this bias, but we also found a significant difference between mothers and fathers on the PRAM-SBD, also suggesting that at the end of the second trimester, fathers feel less attached, connected or bonded with the fetus than mothers. These differences between men and women may disappear at the end of the pregnancy in the third trimester [36], since a growing affection of both parents towards the unborn child during the course of pregnancy has been found in previous studies [49]. Empirical studies support this notion and have shown that prenatal attachment increases during pregnancy [18], although individual scores on prenatal attachment seem to be relatively stable from the first to the third trimester [19]. Especially in the second trimester, fetal growth is more observable and the child begins to feel more real to most women. Ultrasound technology has contributed significantly to recognizing the reality of the pregnancy and the developing fetus [50]. Especially for expectant fathers the effect of ultrasound was stronger for the recognition of the pregnancy and bonding than feeling the baby’s movements and the growing abdomen [51,52]. Although differences between parents may disappear at the end of the pregnancy, Ustunsoz et al. [35] found similar significant gender differences in the third trimester suggesting that men remain less attached and oriented towards their fetus compared to women. Administering the PRAM at different time points during pregnancy may yield more insight into the development of bonding over time.

Parity was found significantly related to bonding as measured by the PRAM, MAAS and PAAS. Primi- and multiparae showed significant different scores on the MAAS and PAAS (sub)scales and also showed different PRAM-SBD scores. However, this latter applies only to women. The PRAM and questions -as included in the MAAS and PAAS- about how often parents think about the fetus and whether they feel affection, confidence, or annoyance by the fetus might be highly colored by postnatal experiences they have with other children and may be affected by having had these experiences before in a former pregnancy. Further studies, however, are needed to substantiate these assumptions.

We further found younger women showing stronger feelings of attachment towards the fetus than older women. This is in accordance with studies of Ustunsoz et al. [35], where younger parents showed more affect and were more preoccupied with the unborn child than older parents. Also a study by McMahon et al. [44], recently showed that women having their first baby when older, appear to have some psychological advantages over younger women, because they are more resilient and better adjusted. This may result in less preoccupation with their pregnancy and the fetus (i.e., as reflected in lower maternal-fetal attachment scores). However, according to meta-analytic studies [47], demographic predictors such as age -with low effect sizes in relation to antenatal attachment measures- are considered as factors that are less useful for strict theory building but merely need to be included in studies as potential confounding factors.

A limitation of this study is that we did not take factors such as mental state of the parents and social support into account, which have been found to affect attachment feelings. Alhusen [53], for example, found that having a supportive spouse positively affected feelings of prenatal attachment. Moreover, in the present study, only pregnant women without medical risk participated. When the pregnancy is high risk and mothers are referred to obstetricians, it may be more difficult for expectant women to cope with their pregnancy. Feelings of attachment towards the fetus may then be adversely affected, when being overwhelmed by one’s own problems [54]. Extension of the evaluation of this measure to clinical and high-risk samples is recommended and may further add to our knowledge of optimal bonding and the usefulness of PRAM-SBD for clinical purposes.

Furthermore, we assumed that a smaller PRAM-SBD is more positive and reflects more attachment. This is also confirmed by our findings. However, we do not know yet whether there is an optimal distance between Baby and Self. In other words, one could argue that the relationship between PRAM and attachment/bonding is not linear, but rather curvilinear. Although we generally expect larger distances to be related to less feelings of attachment or bonding, we need to be cautious by interpreting the results in such as way. One might argue that placing the sticker (i.e., the fetus) too close to the ‘Self’ with a minimal PRAM-SBD is not the same as feeling optimally attached. It rather might reflect strong preoccupation with the child. Because of a lack of norm-scores, we have to be careful with interpreting the PRAM-SBD. In a recent study with very preterm (born <31 weeks gestational age) and moderate preterm infants (born between 32–37 weeks gestational age) [55]), longitudinal analyses revealed that mothers’ PRAM-SBD scores decreased after moderately preterm delivery, whereas decreases in PRAM-SBD scores were observed in both parents after very preterm delivery. As lower PRAM-SBD scores represent stronger feelings of parent-infant connectedness, these findings suggest a higher degree of bonding after premature childbirth. These results were in line with outcome measures operationalized with other instruments, as parents of preterm infants reported less bonding problems on a questionnaire compared to parents of full-terms. These findings may support the hypothesis that in affluent countries with adequate resources, bonding in parents of preterm infants on average may be higher than in parents of full-term infants. Future studies with various clinical populations, in developing countries or in parents with very limited resources, as well as later follow-up measurements, are still needed to clarify the development and process of parent-infant bonding. Moreover, future studies are needed to gain more insight into optimal distances between Self and Baby.

Since it is not possible to conduct item analyses or inter-item consistency measures on a single-item scale [56], no reliability analyses have been performed. Nevertheless, this study showed evidence of convergent validity of the PRAM with other measures of prenatal attachment, even when the response mode varied considerably. Moreover, it could be argued that the significant positive correlation that we found between the PRAM-SBD and the MAAS or PAAS might be indicating the desire of some participants to respond in a socially desirable way. The range of scores (i.e., distance between self and baby), however, indicates that parents felt free to put the sticker close to themselves or at larger distance. Finally, we did not counterbalance the assessment of the M/PAAS and the PRAM and therefore were not able to control for order effects. Future studies using the PRAM with a focus on validity and reliability aspects of this instrument (i.e., discriminant and predictive validity, short-term test-retest reliability) are strongly recommended and are also in progress by our research group.

The clinical usefulness of an instrument further depends on its capacity to identify clinical cases, so that it can be used for screening purposes, to detect parents, for whom the bonding process may not adequately develop. In addition, sensitivity to change is another important asset of a clinical instrument. Can it be used to evaluate the effects of an intervention, aimed to stimulate the bonding process? A recent study by Wittmann et al. [26] showed a similar instrument to be sensitive to change in PTSD patients. We recommend further research in clinical groups.

## Conclusions

The present findings provide support that the PRAM is as a valid, quick, and easy-to-administer instrument of parent-infant bonding. However, further research focusing on its capacity as a screening instrument (to identify parents with serious bonding problems) and its sensitivity to change (necessary for the use in evaluation of intervention studies) is needed, in order to prove its clinical value.

For now, the results showed that the PRAM can be used as an effective instrument of parent-infant bonding in research.

## Competing interests

The authors declare that they have no competing interests.

## Authors’ contributions

The present study was developed by HvB and AV, in collaboration with AM and CV at the department of Developmental Psychology, Tilburg University, Tilburg, the Netherlands. AM and CV were appointed as PhD-students in 2008 and executed the study. All collaborators are considered as co-authors as they have significantly contributed to developing this research, obtaining the data, and writing the manuscript. All authors read and approved the final manuscript.

## Authors’ information

HvB: Department of TRANZO, Tilburg University, Tilburg, the Netherlands, Centre for Infant Mental Health, Dimence, Deventer, the Netherlands and Herlaarhof, Centre for Child and Adolescent Psychiatry, The Netherlands. AM, CV, Department of Developmental Psychology, Tilburg University, Tilburg, the Netherlands. AV: Department of Medical and Clinical Psychology, Tilburg University, Tilburg, the Netherlands.

## Pre-publication history

The pre-publication history for this paper can be accessed here:

http://www.biomedcentral.com/1471-2393/13/138/prepub
